# Social Pre-treatment Modulates Attention Allocation to Transient and Stable Object Properties

**DOI:** 10.3389/fpsyg.2016.01619

**Published:** 2016-10-25

**Authors:** Katalin Oláh, Fruzsina Elekes, Borbála Turcsán, Orsolya Kis, József Topál

**Affiliations:** ^1^Institute of Cognitive Neuroscience and Psychology, Research Centre for Natural Sciences, Hungarian Academy of SciencesBudapest, Hungary; ^2^Institute of Psychology, Eötvös Loránd UniversityBudapest, Hungary; ^3^Cognitive Development Center, Central European UniversityBudapest, Hungary; ^4^Department of Cognitive Science, Budapest University of Technology and EconomicsBudapest, Hungary

**Keywords:** social pre-treatment, communication, change detection, object, location, identity

## Abstract

Increasing evidence suggests that ostensive-communicative signals in social learning situations enable observers to focus their attention on the intrinsic features of an object (e.g., color) at the expense of ignoring transient object properties (e.g., location). Here we investigated whether off-line social cues, presented as social primes, have the same power to modulate attention allocation to stable and transient object properties as on-line ostensive-communicative cues. The first part of the experiment consisted of a pre-treatment phase, where adult male participants either received intensive social stimulation or were asked to perform non-social actions. Then, they participated in a change detection test, where they watched pairs of pictures depicting an array of five objects. On the second picture, a change occurred compared to the first picture. One object changed either its location (moving forward or backward) or was replaced by another object, and participants were required to indicate where the change had happened. We found that participants detected the change more successfully if it had happened in the location of the object; however, this difference was reduced following a socially intense pre-treatment phase. The results are discussed in relation to the claims of the natural pedagogy theory.

## Introduction

The human information processing system is constantly bombarded with an excess of stimuli, of which only a small portion can be effectively processed. Due to the limited capacity of the system, irrelevant stimuli must be filtered out with the help of selective attention processes (Broadbent, 1958).

Although the literature on selective attention mostly deals with low-level visual and acoustic processing, selectivity is not only vital in such cases, but also has a fundamental role in social learning contexts. It has been well-established that human infants’ learning processes are driven to a great extent by observing the behavior of fellow humans and by copying their actions (e.g., Bandura, 1977; Nagell et al., 1993; Gergely and Csibra, 2006, etc.). One of the puzzling and characteristic features of human infants’ and children’s imitative behavior is that it seems to be *faithful* and highly *selective* at the same time (Over and Carpenter, 2012). In learning processes, selectivity can occur in at least two stages. First, learners may observe and encode every aspect of the demonstration, but choose to omit certain elements when re-enacting the action based on different criteria. Second, selection between stimuli may already happen at encoding: since in natural settings, almost any observed situation contains a number of irrelevant (therefore, distracting) elements, learners may perform better if they selectively attend only to the relevant ones from the beginning. The challenge lies in deciding which elements can be considered relevant and which cannot.

According to recent theories, the evolution of the human brain has been greatly determined by our capacity to follow the communicative signals of knowledgeable individuals in making this decision (Csibra and Gergely, 2006, 2011). The Natural Pedagogy theory (Csibra and Gergely, 2006, 2009) proposes that humans are born with a specialized mechanism that makes them sensitive to cues that imply the teaching intentions of others and that prepare them for knowledge acquisition in a social context. These cues include ostensive signals (e.g., eye-contact) that indicate that the other person is about to present relevant information, and referential signals that specify the target object.

It has been demonstrated in a number of studies that even infants are sensitive to such pedagogical cues. For example, Senju and Csibra (2008) have shown that infants take the gaze of a protagonist as a referential action and follow its direction, but only if the action was preceded by ostensive signals. This sensitivity has also been shown to exist at the neural level (Parise and Csibra, 2013). Moreover, the pedagogical context plays an important role in learning situations. Children copy actions more faithfully when the demonstration is placed in a communicative setting than when the demonstration is not accompanied by such cues (e.g., Southgate et al., 2009; Király et al., 2013).

An important prediction of the Natural Pedagogy theory is that the communicative presentation modulates the type of information extracted from a situation. Specifically, while in a non-communicative demonstration people will be more likely to encode episodic information (that is bound to the given situation), in the presence of communicative cues, people will search for information that can be generalized to other contexts as well (genericity bias). This ability of humans to switch to a mode of information processing where they focus on the stable aspects of the environment and make generalizations to kinds is one of the features of the human mind that separate us from other species (Csibra and Gergely, 2011). This allows us to be freed from the bounds of the “here-and-now” and thus lays the ground for a number of higher-order functions, such as symbolic thinking. This prediction has been investigated in a few studies with children. For example, Topál et al. (2008) used the well-known A-not-B error to demonstrate the effects of communication in guiding action interpretation. In this task, children watch an experimenter repeatedly hide a toy in one of two locations. In a subsequent trial, the experimenter changes hiding location but children below the age of 12 months of age generally continue to (mistakenly) search in the original hiding place. Topál et al. (2008) have shown that by eliminating ostensive signals from the demonstration, children’s error rates can be significantly diminished. They suggest that this is due to the fact that with the original procedure (in which the experimenter does not withhold communicative cues, such as looking in the child’s eye, addressing them, etc.), children interpret the ostensive demonstration as referring to information that can be generalized to other contexts (e.g., “the toy belongs in location A”) and this makes them respond the same way in the critical trial as they have in the previous trials. Removing communicative cues from the demonstration eliminates this bias.

Other studies have explored the prediction that follows from the genericity bias, namely that infants and children expect communicatively presented knowledge of an artifact to refer to a kind of object, rather than just the one involved in the presentation. Futó et al. (2010) have shown that communicatively presenting the functions of artifacts led 10-month-old infants to represent the objects in terms of their kind, and that such a category representation even had the power to override the significance of simple visual features. In a study with older children (aged 3 and 4), Butler and Markman (2012) demonstrated a similar effect by showing that children expected object properties to extend to other members of the category following a communicative demonstration of object functions as opposed to following an incidental demonstration.

These results suggest that a communicative demonstration highlights the properties of the object that are relevant for representing object kinds. This may enable observers to focus their attention on and selectively encode kind-relevant information at the expense of ignoring transient object properties. To investigate the effects of communication on selective attention, researchers have used paradigms where participants have to recall two different types of information about objects: their identity and their location (e.g., Yoon et al., 2008). Location is a transient property of an object that may change over time, whereas its identity tends to stay the same. It has been shown that 9-month-old infants retain information about an object’s identity following a communicative demonstration, whereas they are more likely to notice a change in its location after a non-communicative demonstration (Yoon et al., 2008). The bias described above has been investigated in adults as well (Marno et al., 2014). In a change detection paradigm, adults were more likely to notice changes in an object’s location if there were no communicative cues presented whereas they were better at detecting changes in the object’s identity when ostensive-referential cues accompanied the initial presentation of the objects.

Although ample evidence supports the existence of such a genericity bias following communicative demonstrations, the underlying mechanism is not yet fully understood. Marno et al. (2014) suggest that the observed attention modulation effect can possibly be made up of two partly distinct processes. First – at the higher level –, communicative cues may directly alter the interpretation of the observed scenario, which leads to selectively encoding information that is relevant within the framework of the specific interpretation. Second, communicative cues may also exert their effects at a lower level by modulating the functioning of certain neural pathways and thus attuning the nervous system to a specific mode of information processing.

To explore whether and how the latter process may play a part, in this study we investigate whether pedagogical stance can be elicited and sustained over a longer period of time by engaging participants in social interactions that are separated in time from stimulus presentation and thus act as primes. This separation ensures that we only observe the effects of the (potential) low-level processes behind the genericity bias since in such a case, higher level, interpretative processes cannot be engaged because the task and the social cues do not become integrated within one context. Priming effects have been repeatedly demonstrated in the social psychology literature (for a review see Bargh, 2006). Although most of these studies link a specific prime with a highly correlated concept or behavior (e.g., the concept of “rudeness” as prime and subsequently interrupting the interaction partner more frequently, Bargh et al., 1996), in certain cases more general concepts may effectively prime behavior. For example, Over and Carpenter (2009) presented 18-month-old children with photographs of human-shaped figures that evoked a feeling either of affiliation or individuality. In a subsequent task, children who had seen pictures of affiliation helped another person more often than children who had participated in the individuality condition.

The aim of this study, therefore, is to explore whether intensive social stimulation (including an important communicative cue: sustained eye-contact) can exert an effect on information processing that is similar to priming effects. If such effects exist, that would suggest that the observed modulation effects of pedagogical cues not only operate at a higher level of information processing but are at least in part due to low level changes. More precisely, certain cues may trigger changes in the neurohormonal system that prepare the nervous system for a mode of information processing that has adaptive value in social exchanges. One potential candidate that may have a role in regulating social information processing is the neurohormone oxytocin. Oxytocin has been linked to the unique sociality and socio-cognitive skills of humans (Lee et al., 2009). In particular, it has been shown to promote social interactions by reducing social anxiety (Kirsch et al., 2005) and directing attention to socially relevant stimuli (e.g., Guastella et al., 2008). Changes in the level of oxytocin (or other neurohormones) thus may contribute to the observed information processing biases by preparing the nervous system to attend to socially relevant information.

To test this hypothesis, we adapted the change detection task of Marno et al. (2014) to explore whether the human cognitive system can be primed to the specific mode of information processing. Adult male participants, after having received socially stimulating or ignoring pre-treatment, watched pairs of pictures depicting an array of five unfamiliar objects. On the second picture, either the location or the identity of one object was changed and the participant’s task was to indicate where the change had occurred. We followed the methods of Kis et al. (2013) for the pre-treatment phase (alternating eye-contact and tactile stimulation), as they have shown that this kind of social stimulation is effective in modulating social information processing (perception of facial expression) and the effects parallel those of intranasally administered oxytocin, our strongest candidate to mediate the effects. We hypothesized that intensive social stimulation (eye gaze and physical contact) can act as a prime to the pedagogical predisposition through evoking changes in the neurohormonal system and thus such social interactions may have a carryover effect on a subsequent change detection task. In other words, the altered mode of information processing that is observed in a communicative context (paying more attention to the intrinsic features of an object) may also be triggered by social interactions that precede the demonstration of information (and which may not be specifically pedagogical in themselves) by effectively tuning the mind to a specific mode of information processing.

## Materials and Methods

Ethical approval was obtained from the National Psychological Research Ethics Committee (Ref. No. 2011/13). Participants signed informed consent prior to participation. The consent form described the procedure of the experiment, and stated that the purpose of the experiment was to investigate the effect of different types of social stimulation on memory processes.

### Participants

Forty-one adult males between the ages of 18 and 35 (mean: 23.05 years, SD: 2.38 years) participated in the study. Participants were recruited through advertisements in local universities and on various online pages. Participants were randomly assigned to one of the following two conditions: Socially Stimulating (*n* = 21) or Socially Ignoring pre-treatment (*n* = 20).

### Stimuli

**FIGURE 1:**
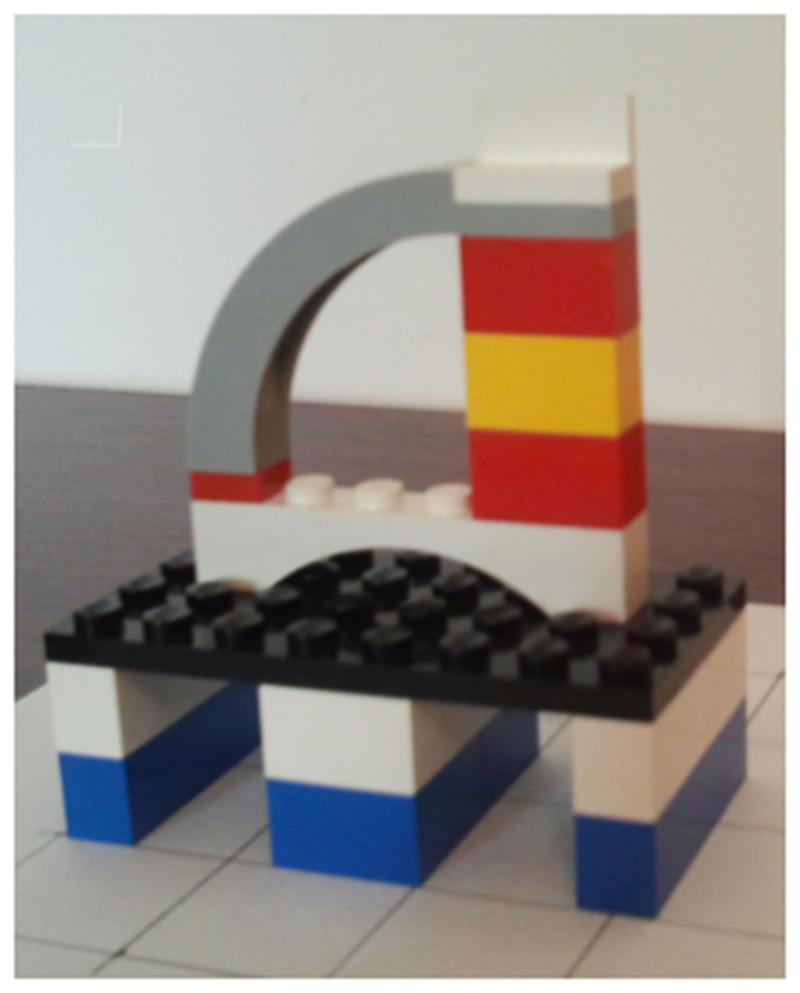
**Example of the objects used as stimuli.** Example of the eight objects used as stimuli. Each lay-out included five of the stimulus objects.

The Stimulus set consisted of 72 pairs of photographs that always depicted an array of five objects, assembled from differently colored Lego bricks (**Figure 1**). The presented objects were selected from a set of eight objects and were placed on a table covered with a yellow-green table cloth. In the first picture of each pair, the objects were arranged in a 3 (rows) × 5 (columns) matrix with each column containing an object in one of the three possible rows. The second picture of each pair depicted the same arrangement as the first one with one change either in the location or the identity of one of the objects. Location change entailed one object moving forward or backward, while in the case of a change in identity, the object was replaced by one that had not been present in the initial picture. We further created three conditions from the total of 72 pairs. 24 pairs were presented neutrally with nothing else present but the table, the objects and a chair behind the table (*Non-referential condition*). The other 48 pairs of pictures included a protagonist sitting behind a table and pointing at one of the objects. The pointing gesture could either correctly signal the object that was about to change (*Referential – Reliable condition* – 24 pairs) or could be misleading (*Referential – Non-reliable condition* – 24 pairs). The referential cue only appeared on the first photograph of each pair; while on the second picture, the protagonist sat looking down without performing any gestures. In each condition, half of the trials included a change in location while the other half included a change in identity (**Table 1**). The following were balanced across conditions and participants: the number of changes that occurred with each object; the number of changes that occurred in each column; the number of times a particular place was referred; the number of misleading and correct signals occurring at a particular place.

**Table 1:** Number of trials in the different conditions.

	Non-referential	Referential-reliable (pointing)	Referential- Non-reliable (deceptive pointing)
Location Change	12 trials	12 trials	12 trials
Identity Change	12 trials	12 trials	12 trials
Total	24 trials	24 trials	24 trials

Stimuli were presented using PsychoPy software on a 12.1″ laptop screen with a resolution of 1366 × 768.

### Procedure

#### Pre-treatment

The first phase of the experiment consisted of the pre-treatment that varied as a function of the condition the participant was assigned to (*Socially Stimulating/Socially Ignoring*). The procedure of the pre-treatment followed that used in the study of Kis et al. (2013) and lasted 6 min. The participant and the experimenter (who was not the person appearing in the photos used as stimuli – see below) were seated next to a table (both on the same side of it), ca. 60 cm from each other. In the Socially Stimulating condition, they were facing each other, while in the Socially Ignoring condition the experimenter turned her back to the participant. The pre-treatment consisted of two alternating 30-second-long phases. In the Socially Stimulating pre-treatment condition, during the first phase, participants were instructed to keep eye-contact with a female experimenter for 30 s. In the second phase, the experimenter pretended to take the participants’ pulse manually, by touching their wrists. The Socially Ignoring pre-treatment followed the same structure; however, in the first phase participants were instructed to fixate on the back of the experimenter’s head rather than looking into her eyes. In the second phase, the participant was told that his pulse would be measured with the help of a pulsometer. Participants could receive feedback about the results of the pulse-taking during the briefing that followed the experiment if they requested it (in both conditions).

#### Change Detection Task

**FIGURE 2:**
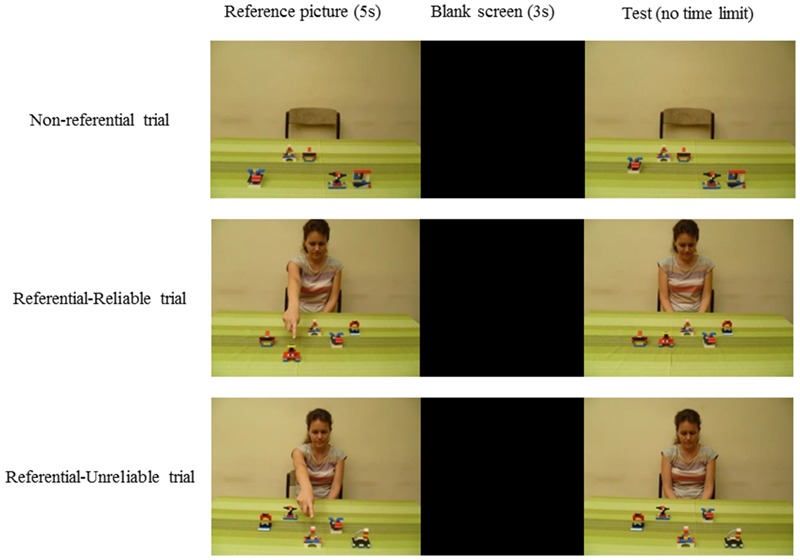
**Experimental design.** Participants saw pairs of pictures depicting an array of novel objects assembled from Lego bricks. The demonstration picture stayed on the screen for 5 s which was followed by a blank screen. The test picture included a change in the location or the identity of one of the objects. The figure shows the different trial types (Non-referential, Referential-Reliable, and Referential-Unreliable).

The change detection task immediately followed the pre-treatment phase. Participants were seated ca. 40 cm from a laptop screen and after a short explanation from the experimenter, the stimulus presentation began. Participants were presented with 72 pairs of photographs (see **Table 1**) in a random order. The first picture stayed on the screen for 5 s; this was followed by a 3-second-long intermission before the test picture appeared. The test picture remained on screen until participants made an answer by pressing a key (**Figure 2**). Before the presentation began, participants were told that they would be viewing pairs of pictures depicting an array of objects and that their task would be to memorize the initial arrangement of the objects. They were also told that the second picture would contain one change compared to the first one: an object would either move forward or backward on the table or it would be changed to an object that had not been present on the previous picture. Participants were asked to indicate in which column the change had occurred after viewing the second picture. This could be done by pressing the number keys 1 through 5. Importantly, they were *not* required to specify the type of change (i.e., location or identity). They were also instructed to ignore the presence of the protagonist and only focus on the task described above.

#### Data Analysis

First, as explorative analysis, we investigated if any characteristics of the trial affected whether the participants’ response was correct or not using Generalized Linear Mixed Model (GLMM) with Binary Logistic Regression. The dependent variable was the correctness of the response (yes/no), the fixed effects were (1) the object’s identity (which one of the eight possible objects changed, 1–8); (2) column changed (in which column has the change occurred?, 1–5); (3) position changed (in which position the object was when the change occurred, front-middle-back); (4) version of change (the object moved forward, moved backward or the identity has changed); (5) trial order (the rank order of the trials, 1–72), and we included the participants’ ID as random factor (as we had 72 data from each participant).

Another Binary GLMM was used to analyze the effects of condition (Socially Stimulating/Socially Ignoring pre-treatment), change type (identity/location), trial type (Non-referential/Referential-Reliable/Referential-Non-reliable) and their interactions on the response success. Moreover, we also analyzed the participants’ latency to respond in each trial (72 data from each participant), using GEE (Generalized Estimating Equations). In this model the fixed effects included condition (Socially Stimulating/Socially Ignoring pre-treatment), change type (identity/location), trial type (Non-referential/Referential-Reliable/Referential-Non-reliable), response success (correct/incorrect), and their interactions. In all three models we used backward selection to remove non-significant (*p* > 0.05) factors from the saturated model. All statistics were performed using IBM SPSS Statistics 22.

## Results

### Contextual Factors that Affect Change Detection

The object’s identity had an effect on participants’ performance [*F*(7,2937) = 4.062, *p* < 0.001], showing that change detection was not equally easy for all objects. The column where the change occurred had a significant effect on the response success as well [*F*(4,2937) = 3.352, *p* = 0.01], participants were more successful when the change occurred in the leftmost column compared to the one next to it (*p* = 0.034), the middle column (*p* < 0.002) and the one next to the rightmost column (*p* = 0.002). The starting position within the column (front-middle-back) also affected the participants’ success [*F*(2,2937) = 12.03, *p* < 0.001], they detected the change more likely when it occurred in the front position compared to the middle (*p* < 0.001) and back (*p* = 0.002) positions. Finally, we have found a significant effect of trial order [*F*(1,2937) = 36.624, *p* < 0.001], participants became more successful as they progressed through the experiment.

### The Effects of Pre-treatment and Change Type on Accuracy

**FIGURE 3:**
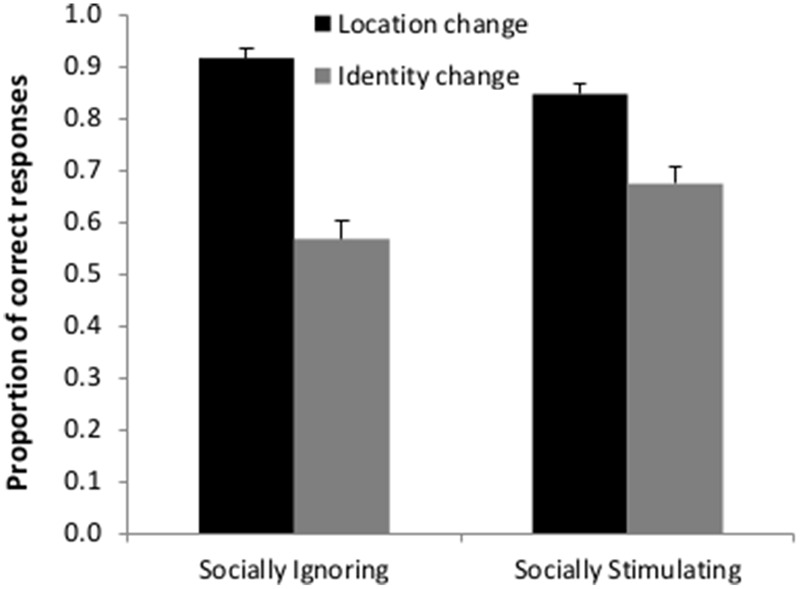
**Detection of location and identity change following a Socially Stimulating or a Socially Ignoring pre-treatment.** The graph depicts the mean proportion of correct answers in the two pre-treatment groups. The results show an interaction between the two factors (type of change and pre-treatment). Participants were better at detecting the change if it occurred in the object’s location; however, the difference between correct choices for the two types of change was smaller following a Socially Stimulating pre-treatment. Error bars represent the upper bound of the 95% confidence interval.

Unlike condition (Socially Stimulating vs. Socially Ignoring pre-treatment, *F*(1,2946) = 0.133; *p* = 0.715), change type (identity or location) had a significant main effect on participants’ performance [*F*(1,2946) = 254.579; *p* < 0.001] with more correct responses in the case of location change (mean proportion (M): 0.852; SD: 0.107) than identity change (M: 0.652; SD: 0.175). Importantly, however, the interaction between condition and change type was also robustly significant [*F*(1,2946) = 31.906; *p* < 0.001] showing that while in both the Socially Stimulating and the Socially Ignoring pre-treatment groups, location change detection was easier than identity change detection, this difference was smaller in the Socially Stimulating pre-treatment group (**Figure 3**). Using pairwise analyses (Least Significant Difference method, GLMM *post-hoc* pairwise contrasts) we have found that the participants in the Socially Stimulating condition made more correct answers in the case of identity change than participants in the Socially Ignoring condition [*t*(2946) = 2.114; *p* = 0.035], while the opposite was true for location change [*t*(2946) = 2.409; *p* = 0.016] with less correct answers in the Socially Stimulating than in the Socially Ignoring condition.

**FIGURE 4:**
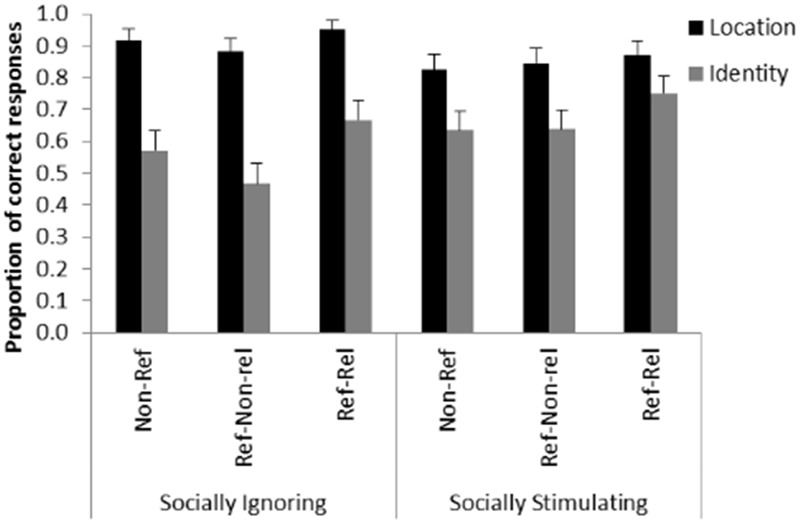
**Detection of location and identity change in the different conditions.** The graph shows the mean proportion of correct responses by experimental group (Socially Ignoring and Socially Stimulating) and condition (Non-referential /Non-Ref/; Referential-Non-reliable /Ref-Non-rel/; Referential-Reliable /Ref-Rel/). Next to the interaction between pre-treatment and type of change, the graph shows that participants were better at detecting changes following a reliable pointing gesture independently of pre-treatment group and change type. Error bars represent the upper bound of the 95% confidence interval.

Trial type also had a significant main effect on performance [*F*(2,2946) = 16.707; *p* < 0.001]. Pairwise analyses revealed that the proportion of correct responses was higher in the Referential-Reliable trials compared to both the Non-referential trials [*t*(2946) = 4.044; *p* < 0.001] and the Referential-Non-reliable trials [*t*(2946) = 5.336; *p* < 0.001]. We found no difference between the Non-referential and the Referential-Non-reliable trials [*t*(2946) = 1.487; *p* = 0.137] (**Figure 4**).

### Response Latency

**FIGURE 5:**
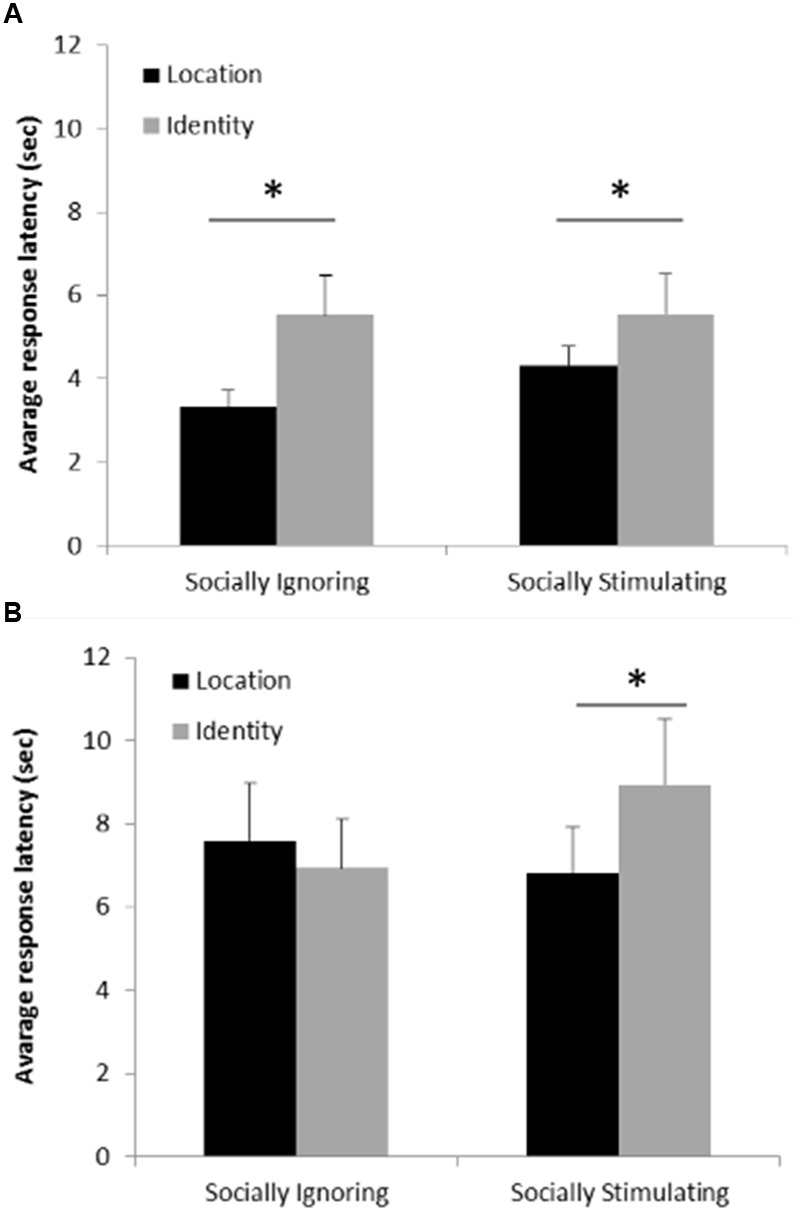
**(A)** Average response latency on correct trials. The graph shows the average response latencies on trials where participants correctly detected the change. Participants responded faster on trials where location change occurred compared to trials with identity change, independently of experimental group. Error bars represent the upper bound of the 95% confidence interval. **(B)** Average response latency on incorrect trials. The graph shows the average response latencies on trials where participants gave incorrect responses. Participants responded faster (made the incorrect choice faster) on location than on identity change trials only in the Socially Stimulating condition. Error bars represent the upper bound of the 95% confidence interval. Asterisk indicates a significant difference.

Analyzing the effects of condition, change type, trial type and response success on participants response latencies, there was no effect of trial type (χ^2^(1) = 2.356; *p* = 0.308 at removal), however we found a significant three-way interaction between condition, change type and response success (χ^2^(1) = 9.549; *p* = 0.002). To further explore and interpret this interaction, we investigated the two types of response (correct/incorrect) separately. These models included only condition, change type and their interaction. In the case of correct responses we found only a main effect of change type (χ^2^(1) = 31.933; *p* < 0.001) showing that participants’ responses were faster in detecting a change in location than a change in identity (**Figure 5A**). In the case of incorrect responses the interaction between condition and change type was significant (χ^2^(1) = 6.146; *p* = 0.013). Pairwise analyses contrasting the conditions revealed no significant difference in the response latency between the change types in the Socially Ignoring condition (*p* = 0.374), while in the Socially Stimulating condition participants answered faster on location change trials than on identity change trials (*p* = 0.012). Pairwise analyses contrasting the change types revealed no significant difference in the response latency between the conditions in the case of location change (*p* = 0.383), while on identity change trials, participants answered faster in the Socially Ignoring condition than in the Socially Stimulating condition (*p* = 0.048). In sum, latencies increased in the Socially Stimulating condition when participants had to make a decision about identity change (and ended up making a wrong choice) (**Figure 5B**).

## Discussion

In this study, we aimed to test the modulatory effects of off-line social cues on the allocation of attention in a change detection task. We adapted the methods of Marno et al. (2014) to examine whether adults are better at detecting changes in an object’s identity or in its location following a phase of socially stimulating or socially ignoring pre-sensitization. We have found that off-line social cues had a similar, though less pronounced, effect on the encoding of different object properties as on-line ostensive-communicative cues in the Marno et al. (2014) study. First, the results show that participants were significantly better at correctly identifying the change if it happened in the location of the object rather than in its identity. A similar tendency was found analyzing the response latencies of participants. However, this main effect was significantly modulated by the type of pre-treatment. The difference between the proportions of correct responses was smaller after participating in a socially intense pre-treatment phase. Although trial order, object identity and position had significant effects on performance, the main results should not reflect such effects as these factors were balanced across conditions.

These results are in great part consistent with the results of Marno et al. (2014) who have found that location change detection was easier in a non-communicative context and that this pattern was reversed in the communicative context. In our study, social pre-treatment did not result in a better performance in the trials that involved identity change; however, it did reduce the difference between the two types of trials. Marno et al. (2014) have argued that ostensive-referential signals do not simply modulate the amount of attention allocated to different objects but qualitatively change information processing about them. In the former case, we would expect to see a general enhancement in performance. However, the results show that while in the case of identity change, improvement could be observed, the modulation effect constituted deterioration on location change trials. Since communicative cues tune the human mind to process generic information, inhibiting the encoding of transient properties can be regarded as adaptive functioning.

Note, that in the study of Marno et al. (2014) this inhibition was only present when the change occurred with the cued object in the communicative condition; however, we found no interaction effect between trial type and any other factor. Thus, while an ostensive-referential context selectively highlighted the durable properties of the socially referred object only, an extensive pre-treatment with social cues generally enhanced the tendency to focus on the generic properties of all of the objects present. Together with this result, we also found that detecting any kind of change was easier if the protagonist correctly signaled the object that was about to change compared to the cases where the pointing gesture was misleading or absent. This suggests that the inability to inhibit attention to directional cues was not dependent on the type of previous pre-treatment. We propose that these results can be explained by the fact that the referential gesture was never directly preceded by any ostensive signals. This finding is consistent with that reported by Marno et al. (2014) with the same paradigm: they failed to show attention shifting effect (from transient to generic properties) of pointing in the absence of ostensive signals. Thus, in our study, pointing may have worked simply as a general attention directing cue (hence the improvement on Referential-Reliable trials) and not as part of a communicative act (which would have led to selectively enhancing performance on identity trials on the referred object). This also sheds some light on the differential effects of ostensive-referential signals and the social pre-treatment applied in our study. While our results suggest that intensive social stimulation in itself prepares the nervous system for a certain mode of information processing, a pedagogical situation will provide a more sophisticated frame for interpretation as the ostensive and referential cues are perceived in an integrated way to point out the information to acquire.

Analyzing the response latencies of participants we found mostly consistent results with the ones for accuracy. When participants answered correctly, they were overall faster to detect the change in location. On the other hand, they had more nuanced tendencies when making errors, showing that participants were slower on identity-change trials only following a socially stimulating pre-treatment phase (making an *incorrect* choice slower for identity trials). Since participants did not have to indicate whether they witnessed a change in location or identity (they simply had to indicate the column where change had occurred), we cannot be sure of the origin of this slowing down effect. When making a wrong choice on identity-change trials, any of the following three options may underlie participants’ behavior: 1, they mistakenly think that a location change had occurred in another location; 2, they mistakenly think that a different object’s identity had changed; 3, they simply make a random guess. Despite this ambiguity in interpreting the data, elongated processing of the stimulus on identity change trials following a socially intense pre-treatment, possibly reflects that participants felt they were closer to the solution in these cases and that is why they spent more time on the stimulus. This would be in line with the general tendency to be more focused on feature information following social stimulation.

The most important question regarding the results is whether the similarity between the present findings and those of Marno et al. (2014) reflect – at least to some degree – the same processes. Marno et al. (2014) suggest that there may be two processes that contribute to the biases (toward predominantly processing information about object identity or location) observed in similar studies (e.g., Yoon et al., 2008). On the higher level, communicative signals may alter the interpretation of the subsequently presented information, prompting the recipient to encode information about object kinds rather than a particular piece. On the lower level, communicative cues may selectively affect the working of certain neural pathways before interpretation happens. Although the exact neural processes underlying the genericity bias (encoding generalizable information about objects following social cues) have not been identified yet, our results support the claim that pre-interpretative levels of information processing play a part in the phenomenon. Since cues separated from the immediate context are unlikely to affect higher level interpretation, we propose that our results can be accounted for by certain changes in the nervous system that precede higher-level processing. As discussed earlier, ostensive and referential signal create a context where they are integrated into a pedagogical episode and the information pointed out in this context will be interpreted as generic. However, this context is not established in the absence of ostensive signals; which means that a different mechanism is responsible for our results. Without any direct measures of the neural correlates of participants’ behavior, we cannot give a description of the mechanism playing a part here. Nonetheless, the results suggest that intensive social stimulation promotes certain – possibly neurohormonal – changes that evoke a bias in information processing that is similar to that observed in a pedagogical situation. Even though the stimulation applied in our study cannot be regarded as strictly “pedagogical”, the neurohormonal changes are likely very similar in the two cases. Previous studies have shown that cues that are often involved in social learning situations facilitate the release of oxytocin in the human body (Feldman et al., 2010), thus this neurohormone was our best candidate to play a part in the low-level process behind the genericity bias. For this reason, the pre-treatment phase followed a procedure that is appropriate to facilitate the release of oxytocin. Although we cannot be sure whether it was the increased level of oxytocin that made people shift their attention toward stable object properties, our results point to the conclusion that the genericity bias is built up of a low and a high level process. This is evidenced by the difference between the conditions in a case where the social stimulation could not create a “pedagogical episode” which would have provided the framework for interpretation.

As Marno et al. (2014) suggest, another possible low-level change responsible for such effects is that communication (and possibly other social cues) facilitates the ventral visual stream (and/or inhibits the dorsal stream) which is responsible for processing intrinsic information necessary to identify an object (Mishkin and Ungerleider, 1982). Studies have shown that information processing in the two streams may be selectively facilitated or inhibited within the situation by the characteristics of the stimuli that people are faced with (Mareschal and Johnson, 2003) and that the functioning of the two pathways may also be selectively impaired in different clinical conditions, such as schizophrenia (Butler and Javitt, 2005).

Importantly, we cannot give a definite answer to the question whether effects observed in our study and that of Marno et al. (2014) reflect the same mechanism or to what extent they overlap. We propose that communicative cues trigger similar neurohormonal changes (given that they are social cues), however, a higher-level, interpretative mechanism ensures that ostensive and referential cues make the recipient of the communication correctly identify the relevant information. This effect is exhibited in the subtle differences of cueing effects in the two studies.

In summary, our study has shown that off-line social cues invite a similar bias in information processing as on-line communicative signals do and the social context in which information is presented can effectively modify how people process a scenario. Namely, intensive social stimulation influences the allocation of attention between transient and stable object properties to facilitate the encoding of stable, generalizable properties.

## Author Contributions

KO, FE, and JT conceived the experiment; OK ran the experiments; BT analyzed the data; KO and BT prepared the first draft of the manuscript and FE, OK, and JT provided feedback. The final version of the manuscript was submitted with the approval of all of the authors.

## Conflict of Interest Statement

The authors declare that the research was conducted in the absence of any commercial or financial relationships that could be construed as a potential conflict of interest.
